# Editorial: Babesia: biology, interactions, and mechanisms of pathogenesis in ticks and its hosts volume II

**DOI:** 10.3389/fcimb.2023.1346960

**Published:** 2023-12-20

**Authors:** Heba Alzan, Yan Li, Haiyan Gong

**Affiliations:** ^1^ Department of Veterinary Microbiology and Pathology, College of Veterinary Medicine, Washington State University, Pullman, WA, United States; ^2^ Parasitology and Animal Diseases Department, National Research Center, Giza, Egypt; ^3^ Tick and Tick-Borne Disease Research Unit, National Research Center, Giza, Egypt; ^4^ Shandong Vocational Animal Science and Veterinary College, Weifang, China; ^5^ Shanghai Veterinary Research Institute, Chinese Academy of Agricultural Sciences, Shanghai, China

**Keywords:** *Babesia*, omics, genome sequence, RNA-seq, serum metabolome, drug design

*Babesia* is a tick-transmitted protozoal pathogen. It can affect animals and humans worldwide, causing various kinds of diseases. For a better understanding of *Babesia* parasite biology, we created the Research Topic “*Babesia*: *Biology, Interactions, and Mechanisms of Pathogenesis in Ticks and Its Hosts Volume II*.” In this Research Topic, five publications were collected, which focused on omics technologies to describe *Babesia* from different aspects, including genome, RNA, metabolites, and computational analysis for drug discovery.

Since both *Babesia bovis* and *Babesia bigemina* are transovarially transmitted by *Rhipicephalus* ticks, Capelli-Peixoto et al. analyzed the differential gene expression that is required for the *Babesia* life cycle in the vertebrate hosts and invertebrate vector using high throughput RNA sequencing. The results revealed similar patterns of gene regulation between the two tick-borne *Babesia* species. Similar to *B. bovis*, the transcription of several *B. bigemina* genes in kinete stages exceeded a 1,000-fold change, and a few of these genes had a >20,000-fold increase. Moreover, through the comparison of genes between the two parasites (*B. bigemina* and *B. bovis*) to the non-transovarially transmitted ones (*Theileria* spp. and *B. microti*), the authors identified the upregulated genes that may be potential markers for transovarial transmission. This work sheds light on the linkage of the *Babesia*-vector-mammalian host, which should improve our understanding of the parasite life cycle and facilitate babesiosis control.

The work of Shen et al. was performed on protozoan parasite *B. microti*, which is the primary cause of human babesiosis. The results demonstrated that the serum metabolome was significantly influenced by acute infection, including perturbations of metabolites in taurine and hypotaurine metabolism, histidine metabolism, and arachidonic acid metabolism. In addition, taurocholic acid, anserine, and arachidonic acid were considered potential candidates of serological biomarkers for diagnosing *B. microti* infection at the acute stage. Those metabolite candidates could be further examined for their role in disease complexity. The work provided new insights into the mechanisms involved in systemic metabolic changes that occur during *B. microti* infection.

The genome of *B. ovis* was analyzed by Yamagishi et al. in order to help selection of diagnostic markers, drug targets, and antigens for vaccine development, which have not been available so far. In this work, a draft genome sequence with a size of 7.81 Mbp and 3,419 protein-coding genes was explored in *B. ovis*, which was isolated from an infected sheep in Turkey. It consisted of 41 contigs with an N_50_ of 526 Kbp and 259 orthologs, which were identified among eight *Babesia* spp., *Plasmodium falciparum*, and *Toxoplasma gondii*. On the basis of the orthologs, *B. ovis* is the closest to *B. bovis*. Moreover, there were 43 *ves* genes in *B. ovis* predicted by the Hidden Markov model (hmm model), which formed a discriminating cluster to other *ves* multigene families in *Babesia* spp but showed certain similarities to those of *B. bovis*, *B. caballi*, and *Babesia* sp. Xinjiang, which was consistent with the phylogeny analysis. Additionally, this comparative genomic analysis of *B. ovis* and *B. bovis* presented uniquely evolved genes in these species, which could contribute to parasitic adaptation.

*Babesia aktasi* n. sp., a new *Babesia* species/genotypes, is highly prevalent in goats from Turkey’s Mediterranean region. In this Research Topic, Ozubek et al. investigated the pathogenesis of *B. aktasi* infection in immunosuppressed and non-immunosuppressed goats. As described, the presence of parasites in the blood of immunosuppressed goats was detected 4–6 days post-infection and was accompanied by fever and increasing parasitemia. Goats that succumbed to acute disease exhibited severe clinical signs, such as anemia, hemoglobinuria, and loss of appetite. However, milder clinical signs were observed in the goats that survived. In contrast, piroplasm forms of *B. aktasi* with low (0.01–0.2%) parasitemia were observed in the blood within 2–5 days of inoculation in the non-immunosuppressed group. Although these goats showed a loss of appetite, typical signs of babesiosis were absent except for an increase in body temperature. The study provided basic information for the development of effective prevention and control strategies against babesiosis in small ruminants. However, further research is required to investigate the pathogenicity of *B. aktasi* in various goat breeds, other potential hosts, the vector ticks involved, and its presence in natural reservoirs.

*B. microti* can infect both animals and humans, and the current therapeutic options are limited. Drug resistance is also a concern. In order to find new effective drugs against *B. microti*, Akash et al. used computational drug design approaches to analyze nine natural compounds for their potentiality, and two of them, Vasicinone and Evodiamine, were identified as the most promising drugs. Optimization of the ligand structures was performed using density functional theory, molecular docking, molecular dynamics simulations, quantum mechanics such as HOMO–LUMO, drug-likeness, and theoretical absorption, distribution, metabolism, excretion, toxicity (ADMET), and pharmacokinetics. Both drugs showed the highest binding energy and anti-parasitic activity against the *B. microti* lactate dehydrogenase apo form. It was suggested that the molecules mentioned be tested experimentally at various levels, including wet lab, pre-clinical, and clinical, to assess their value.

This Research Topic has presented the application of many omics technologies, including genomics, transcriptomics, metabolomics, and computational analysis. Utilizing these technologies, we can obtain new information on potential vaccine or drug targets and a unique insight into *Babesia*’s biology, which in the end may provide us with new ideas for parasite control.

## Author contributions

HA: Writing – original draft, Writing – review & editing. YL: Writing – review & editing. HG: Writing – original draft, Writing – review & editing.

